# The value of referral information and assessment – a cross sectional study of radiographers’ perceptions

**DOI:** 10.1186/s12913-022-08291-w

**Published:** 2022-07-09

**Authors:** Catherine Chilute Chilanga, Hilde Merete Olerud, Kristin Bakke Lysdahl

**Affiliations:** Department of Optometry, Radiography and Lighting Design, Faculty of Health and Social Sciences, University of South-Eastern Norway USN, Pb 235, 3603, Kongsberg, Norway

**Keywords:** Referral and consultation, Radiology department hospital, Patient safety, Health services research

## Abstract

**Background:**

Radiology professionals are frequently confronted with referrals containing insufficient clinical information, which hinders delivery of safe and quality medical imaging services. There is however lack of knowledge on why and how referral information is important for radiographers in clinical practice. This study explores what purposes referral information is used/ useful for the radiographers, and the benefits of involving them in assessing referrals.

**Methods:**

A cross sectional study was conducted of radiographers recruited through the International Society of Radiographers and Radiological Technologists (ISRRT) networks. A questionnaire was developed and distributed consisting of 5-point Likert scale questions on a) use/usefulness of referral information for 12 listed purposes and b) the benefits of radiographers assessing referrals for 8 possible reasons. The questionnaire was validated using a test–retest reliability analysis. Kappa values ≥0.6 were accepted. SPSS software was used for data analysis and chi-square tests to determine associations between using referral information and background variables.

**Results:**

Total respondents were 279 (*n* = 233 currently in clinical practice and *n* = 46 in other positions). The participants in clinical practice ranked high all 12 listed purposes for use of referral information, and all except one received ≥60% ‘frequent’/‘very frequent’ responses. Use for patient identification purposes received the highest score (97% ‘frequently’/‘very frequently’ responses), followed by ensuring imaging of the correct body region (79% ‘very frequently’ responses). Radiographers not currently working in clinical practice ranked the ‘usefulness’ of listed items similarly. Significant associations between frequent use of referral information and education level were not observed, and only three items were significantly associated with modality of practice. All items on benefits of radiographers assessing referrals received ≥75% ‘agree’/‘strongly agree’ scores. The items ranked highest were promotes radiographers’ professional responsibility and improves collaboration with radiologists and referring clinicians, with 72 and 67% strongly agreed responses, respectively.

**Conclusion:**

Radiographers use referral information frequently for several purposes. The referral information is needed for justifying and optimising radiological procedures, hence crucial for ensuring patient safety and high-quality services. This further emphasis why radiographers perceive several benefits of being involved in assessing the referral information.

## Background

When requesting a radiological procedure, the referring clinician sends a referral (request) form containing the patients’ relevant clinical information to the radiology department [1]. This information is vital for patients’ quality care and utilisation of services in radiology departments [2, 3]. The radiologists and radiographers assess the referral information to determine whether the requested radiological examination is warranted and appropriate to confirm or rule out diagnosis of a given condition or disease [4]. Selecting the most appropriate examination prevents patients’ exposure to unnecessary radiation doses in instances where ionising radiation is used [5]. The patients’ safety when conducting radiological examinations is also dependent on the information in the referral. The radiologists and radiographers will need information about the patients’ identity [2], general physical condition and pre-existing illnesses to ensure safety during the procedure [6]. This could be particularly critical when the procedure requires using contrast-media to better visualise anatomy, [7] as contrast media is known to cause adverse effects in patients with certain pre-existing medical conditions [8–11].

The referral information determines patient and procedure information such as radiographic patient positioning, procedure projections and exposure parameters [6, 12]. High quality referral information also enables better radiologist – radiographer communication on visualisation of pathology and suitable radiographic technique to employ to obtain imaging of diagnostic value [13]. The radiographers could further liaise with the radiologists on acceptable images, taking account of the patients’ condition. The radiology report sent to the referring clinician of the outcome of the procedure is also influenced by the referral information. Studies show that referrals of high information quality improve image interpretation accuracy, clinical relevance, and reporting confidence for the radiologists [14]. The referral information is therefore useful for verifications about a patient and procedures [15], and procedure decision-making [6, 16] along the medical-imaging care continuum.

Referral information accordingly influences the quality of the outcome of the services provided in radiology departments. Inadequate referral information is reported as a cause of false positive diagnosis and incidental findings in medical imaging [17, 18], which instigates futile patient follow-up investigation and treatment, and consequently leading to unnecessary overuse of health resources and services [18]. Scheduling of the radiology examination according to priority or urgency is effectively conducted using the information on the referral [16]. The referral quality therefore potentially affects practicing according to healthcare priority setting criteria.

Despite the many indications outlined above on the value of referral information there is little evidence of why and how this is important in clinical practice for radiographers. This study aimed to map the value of referral information and assessment from the perceptive of radiographers working in clinical and non-clinical settings. The objectives were two-fold; to explore a) for what purpose the radiographers make use of referral information or consider it useful and b) the possible benefits of involving radiographers in assessing referrals.

## Methods

This research is the second part of a larger study on radiographers’ involvement in the process of assessing imaging referrals. The first part of the study analysed the radiographers’ actions and challenges when confronted with unjustified radiology referrals and the paper [19] provides more detail on methods.

### Study settings

A cross sectional study was conducted of radiographers internationally who follow activities organised by the International Society of Radiographers and Radiological Technologists (ISRRT). The ISRRT is the professional organisation representing radiographers globally and its mission is to improve the standards of delivery and practice of diagnostic imaging and radiation therapy worldwide [20]. The target group were radiographers currently working in clinical practice in various imaging modalities (clinical radiographers) and those not in clinical practice but have clinical experience in diagnostic radiography (non- current clinical radiographers i.e. radiography administrators, researchers and educators).

### Participant recruitment and data collection

An online survey was distributed using a web form (‘Nettskjema’) [21] for 5 months, initially in April 2020 and between September and December 2020. The recruitment of participants and data collection was conducted using ISRRT networks (see [19]). Non-probabilistic, convenience sampling methods were employed to collect the data. The targeted sample population was selected because it constitutes of radiographers who are assumed to be active in the profession and familiar or well orientated with practice regulations in their respective countries.

### Questionnaire

A questionnaire was piloted and validated using a test–retest reliability analysis. In the first part of this section of the study, questions about perceived usefulness of referral information were asked. The questions were phrased slightly different for radiographers working in clinical and non-clinical settings. After the statement ‘Information in the referral can be useful for a number of reasons. The radiographers working in clinical setting were asked to rate how often they make use of the referral information for 12 listed number of purposes, while radiographers not currently working in a clinical setting were asked to rate their agreement on the usefulness of the same 12 purposes. A five-point Likert response scale was used in both cases ‘Very frequently, Frequently, Occasionally, Rarely, Never’ and ‘Strongly agree, Agree, Undecided, Disagree, Strongly disagree’, for clinical radiographers and non- current clinical radiographers respectively.

For the second main question, all the participants were asked to rate their agreement (scale: strongly agree, agree, undecided, disagree, strongly disagree), on possible benefits of involving radiographers in assessing radiology referrals. A set of 8 possible benefits were listed. The background section included demographics and professional characteristics of the participants.

### Data analysis

The data was analysed using IBM SPSS statistical software version 26. Descriptive analysis was used to show frequency in percentages. The data was split in to 2 cohorts: radiographers in clinical and non-clinical settings to compare variations in responses between the two groups. To analyse difference in how subgroup of clinical radiographers reported their use of referral information, the scales were dichotomised into frequently to never (1) and very frequently (2), based on the response pattern. A chi-square test of independence was used to determine association between the clinical radiographers perceived use of referral information, with the independent variables: dichotomised education level (Bachelor’s degree/equivalent versus master/PhD degree), and 3 split modality of practice (Conventional radiography versus One advanced modality which included CT, MRI, Ultrasound, Mammography or Nuclear medicine, versus Multiple modalities). A *p* value ≤0.05 was considered statistically significant.

### Ethics statement

Ethical approvals were obtained from the Norwegian centre for research data (NSD) reference number 472337 in Norway. All the participants consented to the study through the online portal.

## Results

### Respondents and settings characteristics

The total number of respondents were 279 (*n* = 233 clinical radiographers, *n* = 46 non-current clinical radiographers), as in Table 1. Most of the respondents where from Asia (Indonesia/Taiwan) (28%), United Kingdom (UK) (23%), Scandinavia (Norway/Denmark) (12%), and Australia (11%). The mean age was 38 years. A total 74% of the participants had education level at bachelor’s degree or equivalent. Modality of practice of the participants was reported as follows; 35% conventional radiography, 32% one advanced modality and 33% multiple modalities.

**Table 1:** Demographic and professional characteristics of respondents (*N* = 279)

Sample characteristics		Non-current clinical radiographers^a^	Clinical radiographers	Total responses
		*n* (%)	*n* (%)	*n* (%)
**Participants**		46 (100)	233 (100)	279 (100)
**Continent/** **country**	Asia (Indonesia/Taiwan)	17 (37)	60 (25.8)	77 (27.5)
	United Kingdom (UK)	10 (21.7)	54 (23.2)	64 (22.9)
	Scandinavia (Norway/Denmark)	3 (6.5)	30 (12.9)	33 (11.8)
	Australia	3 (6.5)	28 (12.0)	31 (11.1)
	Canada	2 (4.3)	10 (4.3)	12 (4.3)
	African countries^b^	3 (6.5)	19 (8.2)	22 (7.9)
	Other countries^c^	8 (17.2)	32 (13.7)	40 (14.3)
**Gender**	Male	26 (56.5)	105 (45.1)	131 (46.9)
	Female	20 (43.5)	128 (54.9)	148 (53.1)
**Age (years)**	< 30	8 (17.4)	63 (27.2)	71 (25.5)
	30–44	19 (41.3)	108 (46.6)	127 (45.5)
	45+	19 (41.3)	61 (26.3)	80 (28.7)
**Education level**	PhD	8 (17.4)	8 (3,4)	16 (5.7)
	Master	13 (28.3)	43 (18.5)	56 (20.1)
	Bachelor+ equivalent	25 (54.3)	182 (78.1)	207 (74.2)
**Modality of practice**	Conventional radiography	18 (39.1)	80 (34.3)	98 (35.1)
	One advanced imaging modality^d^	15 (32.6)	75 (32.2)	90 (32.3)
	Multiple modalities	13 (28.3)	78 (33.5)	91 (32.6)

^a^ Radiographers not currently working in clinical practice included those in administration, research, education or other

^b^African countries; majority of respondents from Rwanda

^c^Other countries included Bangladesh, Cambodia, China, Estonia, Germany, Greece, Guyana, Ireland, Italy, Myanmar, Nepal, Netherlands, New Zealand, Palestine, Philippines, Singapore, Sultanate of Oman, USA, Vietnam

^d^Advanced modality included CT, MRI, Ultrasound, Mammography or Nuclear medicine

### Use/usefulness of referral information

**Fig. 1:**
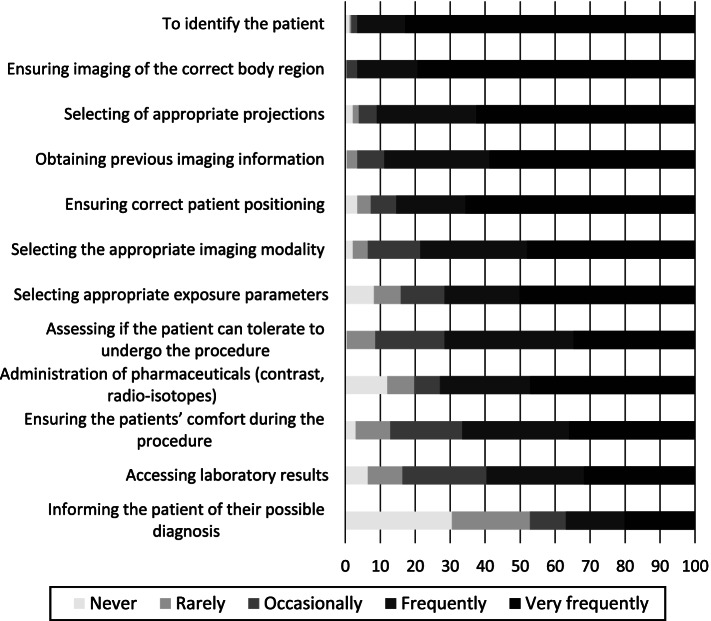
Clinical radiographers frequent use of referral information for tasks in clinical practice. Items are arranged by mean values

**Fig. 2:**
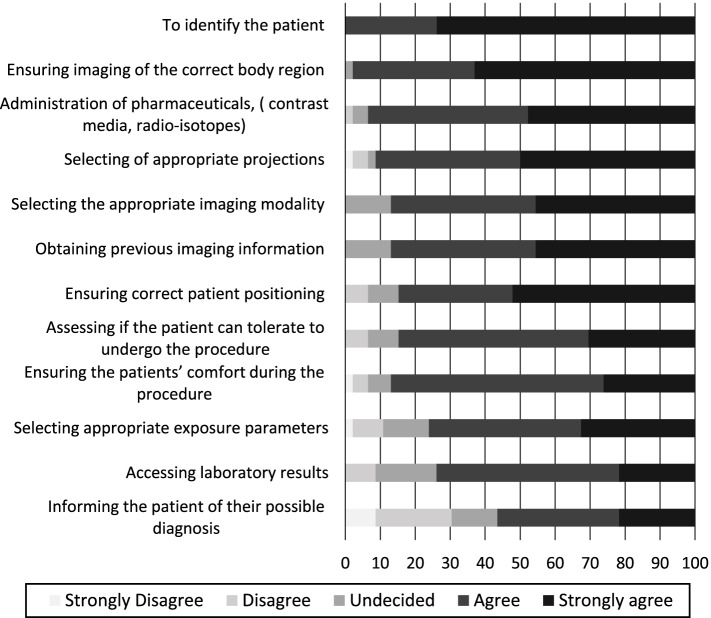
Not-current clinical practice radiographers’ agreement of use of referral information. Items are arranged by mean values

The radiographers in clinical practice reported to very frequently use information in the referral for a variety of reasons (Fig. 1). The radiographers not-currently in clinical settings were also mostly in agreement to the usefulness of the referral information as reported by those in clinical practice (Fig. 2). Details on both groups’ responses follows subsequently.

### Clinical radiographers

Some of the reasons for use of the referral information concerns verification of the patient, which all received high scores. The clinical radiographers rated using the referral information for patient identification highest with combined score of ‘frequently’/‘very frequently’ responses of 97%. Using the referral information to ensure imaging of the correct body region was rated quite high at 79% ‘very frequently’ used responses. This was followed by scores ‘very frequently’ using the information for obtaining previous imaging information (59%).

Other reasons for using the referral information for processes are related to patient positioning, where a rank of ‘very frequently’ responses was obtained for ensuring correct patient position (66%) and selection of appropriate projections (63%). The lowest rank related to patient positioning aspects was ‘very frequently’ to use the information for ensuring the patients’ comfort during the procedure (36%) and assessing if the patient can tolerate to undergo the procedure (35%).

A third group of items concerned use the referral information for procedure decisions, were the highest number of ‘very frequently’ used responses was given for selecting the appropriate exposure parameters, selecting the appropriate imaging modality, and administration of pharmaceuticals (such contrast media, radioisotopes) as 50, 48 and 47% respectively. The lowest rank in this category was ‘very frequently’ using the information in accessing lab results, rated by 32% of respondents.

The overall lowest score was obtained for using the referral to inform the patient of possible diagnosis stated, with 63% ‘never/rarely/occasionally’ responses.

A chi square test performed (Table 2) showed significant association between a few of the variables (items) on the purposes of radiographers’ frequent use of referral information and modality of practice. No significant associations were observed between the use of referral information in the listed items and the dependent variable education level.

**Table 2:** Radiographers’ reported use of referral information^a^ for different purposes^b^ associated with modality of practice

	Modality				
Purpose of use of referral information	Conventional	Advanced	Multiple	Chi square values	*p* values
Access laboratory results	16%	41%	43%	15.808 (df)2	< 0.001
Administration of pharmaceuticals i.e. contrast agents or isotopes	25%	35%	41%	9.614 (df)2	0.01
Selecting of the appropriate imaging modality	31%	27%	42%	7.195 (df)2	0.01

^a^ Percentages of ‘very frequent’ use scores are displayed

^b^ Only the purposes of use items (from the list of 12) with statically significant association are displayed

### Non-current clinical practice radiographers

A similar rating in agreement on usefulness of referral information by the non-current clinical radiographers was observed (Fig. 2). The radiographers not currently working in clinical practice ranked high the usefulness of referral information for patient verification as follows; combined ‘agree’/‘strongly agree’ on patient identification (100%) and ‘strongly agreed’ on ensuring imaging of the correct body region responses (63%). However, agreement as useful to obtain previous imaging information was ranked lower by the non-current clinical radiographers, 46% ‘strongly agreed’ compared to ‘very frequent’ used score stated by 59% clinical radiographers.

Using the referral information for processes of patient position was rated ‘strongly agreed’ for ensuring correct patient position 52% and selecting the appropriate projections 50%. Using the referral information for assessing if the patient can tolerate to undergo the procedure and ensuring the patients’ comfort during the procedure were ranked low in this category as 30 and 26% respectively.

In procedure decisions, the non-current clinical practice radiographers rated use for administration of pharmaceuticals and selecting the appropriate imaging modality as 48 and 46% ‘strongly agreed’ responses respectively. In this category using the information for ‘selecting the appropriate exposure parameters’, and ‘accessing lab results’, was ranked lowest as 33 and 22%, ‘strongly agreed’ responses. Use of the referral information in ‘informing the patient of possible diagnosis’ was however rated higher compared to the clinical radiographers, with combined ranked as useful ‘strongly agreed’/‘agreed scores’ 57% (non- current clinical radiographers) versus 37% ‘very frequent’/‘frequent’ used scores’ (clinical radiographers).

### Benefits of involving radiographers in referral assessment

**Fig. 3:**
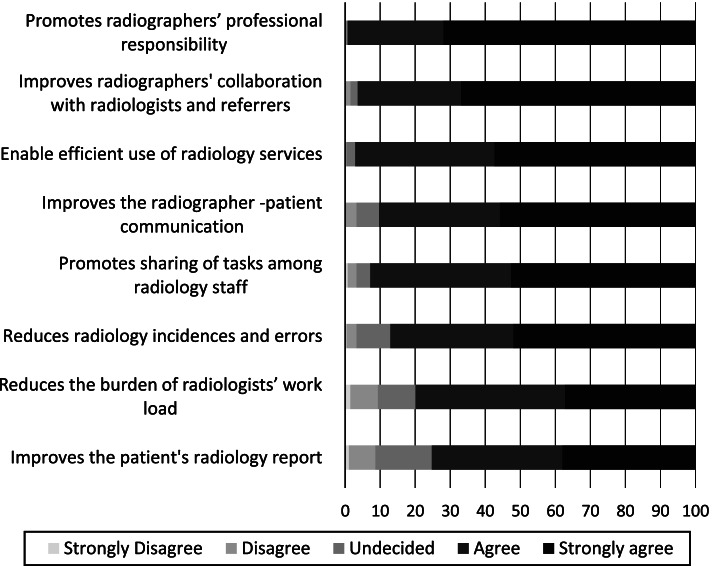
Radiographers’ perceived agreement of benefits of their involvement in assessing referrals. Items are arranged by mean values

In general, the clinical and non-current clinical radiographers provided similar responses on the benefit of involving radiographers in referral assessment. All the listed items (Fig. 3) contained benefits to the patients in shape of quality of care and services, some directly and others more indirect originating from benefits of the healthcare professionals providing the care and services. In total these categories are ranked similarly. Among the items related to direct benefits for quality of care and services, the high ranked were ‘promotes radiographers’ professional responsibility’, ‘enables efficient use of radiology services’, ‘improves the radiographer-patient communication’, and ‘reduces incidences and errors’, receiving 72, 57, 56 and 52% ‘strongly agreed’ responses respectively. Improves the patients’ radiology report was low ranked (‘strongly agreed’ responses by 38% radiographers).

In the category of benefits to the healthcare professionals, the items that ranked high were ‘improves radiographers’ collaboration with radiologists and referring clinicians’ and ‘promotes sharing of tasks among radiology staff’, with 67 and 53% ‘strongly agree’ responses respectively. The lowest score in this category was ‘reduces the burden of the radiologists’ workload’ (37% ‘strongly agree’ responses).

## Discussion

Our study shows radiographers in various imaging modalities frequently making use of referral information for several activities across the imaging care continuum to manage the patient. The crucial value of referral information may not be well known outside the radiology environment. This lack of awareness may be one of the reasons why referrals lacking vital clinical information seems to be a persistent problem [22–25]. Our study shows that radiographers need proper referral information to ensure patient safety, high quality care and services in radiology departments.

### Use of referral information for radiographers’ clinical practice

Our study shows radiographers using the referral information to identify the patient, verify information about the patient and the procedure. Almost all the respondents in our study stated the referral information to be very useful to identify the patient. Ensuring that the radiological procedure is delivered to the correct patient is the starting point of patient safety [15]. The participants further ranked high using the referral information for correct patient position and selection of appropriate radiographic projections which ensures that imaging of diagnostic value is obtained and enable an accurate diagnosis. Patient identification and ensuring the appropriate imaging procedure are selected and conducted optimally further adhere to the two core principles of radiation protection in medicine termed ‘justification’ and ‘optimisation’. The justification principle affirms that the benefits of medical imaging for patients should outweigh the radiation risks and other possible risks [5]. This entails that the radiographers evaluate the referral information against the requested radiological procedure and ensure that the correct imaging modality is selected before performing the procedure. This further ensures that the patient is not exposed to unnecessary risks due to the procedure. Optimisation ensures that radiation doses when used are kept as low as reasonably achievable (ALARA), and imaging of diagnostic value is obtained [5]. During optimisation the imaging procedure, doses, parameters, use of contrast media, and other drugs must be adapted to the individuals’ specific clinical question [7]. Optimisation of procedures will therefore require that the radiographers select the most appropriate radiographic projects and exposure parameters to ensure that the procedure is optimally conducted with the minimum possible radiation exposures to the patient.

Using the referral information for assessing laboratory results and administration of pharmaceuticals is observed in our study mainly in clinical radiographers working in advanced imaging modalities. This was anticipated as most advanced imaging procedures use contrast medium which causes some adverse reactions in certain patients [26]. For example, checking of laboratory results such as blood tests that show estimated glomerular filtration rate can determine kidney function status in patients receiving contrast enhanced imaging [11], and ensures safety use of contrast media during imaging procedures.

In the chi square test analysis in our study, no significant associations were observed between the items on radiographer’s use of clinical information and the dependent variable education level. This indicates that referral information is frequently used by all radiographers’ and at all levels in clinical practice. Our results support those by Lundvall, et al. [27] that states that the radiographers’ professional work and responsibilities in image production involves a process of planning, producing the images, and evaluation, where one of the main features of their professional work is patient safety.

### Benefits of radiographers’ assessing referrals

Our study show that the radiographer’s assessing referrals directly or indirectly facilitates provision of quality care and services in radiology departments. First, the respondents in our study ranked highly that radiographers’ assessing referrals improves professional collaboration with radiologists and referring clinicians and promotes sharing of professional tasks. This indicates that radiographers assessing referrals has benefits for the professionals working within the patients’ referral pathway, which indirectly enhances quality of care and health services. Interprofessional collaborative practice occurs when professionals with different backgrounds work together to deliver the highest quality of healthcare [28]. This provides platforms for better professional communication and teamwork which further support quality patient management across the care pathway [29]. Professional task sharing does not only assist with efficient distribution and organises of work tasks, but also facilitates transfer of knowledge and skills among professions. Knowledge sharing among the radiology professionals is reported to assist with professional development and creates a supporting environment for the radiographers [30]. Supporting environments are further reported to increase job satisfaction of healthcare professionals [31], in turn facilitating provision of quality care.

Second the respondents in our study ranked highly that radiographers assessing referrals promotes professional responsibility. Professional responsibility in healthcare relates to how one performs their work based on ethical values and expected professional standards [32]. Professional responsibility therefore promotes commitment to ensuring quality care. The third factor the respondents ranked high for benefits of radiographers assessing referrals in our study was that it enables efficient use of radiology services. Our findings are supported by Sheth et al. [33], that report that not only does radiographers’ involvement in assessing referrals improve patients’ safety and experience, but also provides an efficient workflow in radiology department. The other benefits to radiographers assessing referral ranked high in our study included, improves the radiographer-patient communication, and reduces incidences and errors. Studies show that through patient communication, radiographers gather vital information about the patient which adds to the patient’s clinical history and is valuable for overall health management [27]. This further reduces incidences and errors [6] and improves the justification and optimisation processes [34]. Ensuring occurrence of justification and optimisation in radiology departments further prevents over-utilisation of radiology resources as unwarranted and repeated imaging is avoided and high- quality imaging of diagnostic value is provided.

### Strength and limitations of study

This study had some limitations. The number of participants were quite low as expected of online survey and the recruitment process. In addition, language contributed to non-responses as the survey was only in English. The difference in organisational processes in radiography departments and country practice legislations could have influenced the radiographers’ responses on how they make use of referral information within their respective institutions. A focused study of individual centres in selected countries could provide better detail on ways and differences radiographers use the referral information. The analysis of responses in the study were however based on expected or required standard practice. The responses on the benefits to assessing referral could be biased towards the radiography profession as the included sample group were only radiographers. The recruitment process indicates that the sample group are not representative for radiographers world-wide, which is an obvious limitation. On the other hand, this sample of radiographers are assumed to be well versed in the profession and assumed to be quite knowledgeable and experienced about the current and expected practices in their various clinical practices and respective countries. This could have strengthened our findings.

## Conclusion

Information in the referral is vital for radiographers’ clinical practice and is used frequently for several purposes. The referral information is needed for justifying and optimising radiological procedures, hence crucial for ensuring patient safety and high-quality care and services. It is therefore vital that radiographers are trained to systematically evaluate and supplement referral information in clinical practice. Radiographers’ involvement in assessing referrals further promotes provision of quality professional work based on ethical values and standards.

## Data Availability

The datasets generated during and/or analysed during the current study are available from the corresponding author on reasonable request.

## Abbreviations

ALARA: As Low As Reasonably Achievable
CT: Computed Tomography
ISRRT: The International Society of Radiographers and Radiological Technologists
MRI: Magnetic Resonance Imaging
